# Assessment of Temporary Community-Based Health Care Facilities During Arbaeenia Mass Gathering at Karbala, Iraq: Cross-Sectional Survey Study

**DOI:** 10.2196/10905

**Published:** 2019-10-04

**Authors:** Faris Lami, Inam Hameed, Ali Arbaji

**Affiliations:** 1 Department of Community and Family Medicine College of Medicine University of Baghdad Baghdad Iraq; 2 Karbala Directorate of Health Iraq Ministry of Health Karbala Iraq; 3 Private Practitioner Amman Jordan

**Keywords:** Arbaeenia mass gathering, community-based health care, Iraq

## Abstract

**Background:**

Arbaeenia mass gathering (MG) in Karbala, Iraq, is becoming one of the largest MGs in the world. The health care infrastructure in Iraq is inadequately prepared to serve the health needs of the millions of pilgrims.

**Objective:**

This study aimed to describe the temporary health care facilities installed and run by the local community to provide health care services to Arbaeenia pilgrims in Karbala, Iraq.

**Methods:**

A survey was conducted in all community-based health care facilities located along part of Najaf to Karbala road within Karbala governorate. A structured questionnaire was answered through an interview with the workers and direct observation. Data were collected on staff profile, type of services provided, use of basic infection control measures, medical equipment, drugs and supplies, and the most commonly encountered medical problems.

**Results:**

The total number of health care facilities was 120, staffed by 659 workers. Only 18 (15.0%, 18/120) facilities were licensed, and 44.1% (53/120) of the workers were health professionals. The health care workers provided different services including dispensing drugs (370/1692, 21.87%), measuring blood pressure and blood sugar (350/1692, 20.69%), and caring for wounds and injuries (319/1692, 18.85%). Around 97% (116/120) health facilities provided services for musculoskeletal disorders and only 16.7% (20/120) provided services for injuries. The drugs available in the clinic were analgesics, drugs for gastrointestinal and respiratory diseases, and antibiotics, with an availability range of 13.3% to 100.0%. Infection control practices for individual protection, environmental sanitation, and medical waste disposal were available in a range of 18.1% to 100.0%.

**Conclusions:**

Community-based health care facilities experienced a profound shortage of trained human resources and medical supplies. They can significantly contribute to health services if they are adequately equipped and follow standardized operation procedures.

## Introduction

### Background

Mass gathering (MG) is the assembly of a large organized or unorganized population in a limited space for a specified period, which often strains available resources and services [1]. The World Health Organization (WHO) also defines MGs as “events attended by a sufficient number of people to strain the planning and response resources of a community, state or nation” [2]. The purpose of the assembly is often for a religious, sport, or social event.

MGs occur frequently around the world and pose a whole spectrum of challenges on local country systems. The public health systems can be compromised during MGs if not well prepared even in countries with appropriate resources to prevent and control endemic diseases [3]. Regardless of the type of MGs, various health problems were reported among MG participants and local communities who are at risk of acquiring infectious diseases or injuries [4-8]. Proper planning before the event can help to prevent causalities and health problems. Countries with abundant resources often develop disaster preparedness plans to address MGs; however, many developing countries cannot afford to allocate adequate resources for such events and may seek assistance from other countries [9].

The provision of basic health care and emergency services is essential for the safety of MG attendees. In Iraq, the task of providing these services is under the authority of the Ministry of Health (MOH).

In Iraq, millions of people gather annually on the 20th day of the lunar month of Safar in Karbala for the remembrance of Arbaeen of Imam Hussain from different countries, mainly from Iran, Afghanistan, Pakistan, and Arab Gulf countries, in addition to several millions of people from most provinces of Iraq walking for hundreds of kilometers en route to Karbala (Karbala is located 100 km southeast of the capital, Baghdad, with a population of approximately 1.2 million inhabitants) over many days that make them at risk of airborne, foodborne, and waterborne diseases and injuries. The Arbaeenia MG is expected to increase in size once the political and security situation of the country stabilizes, as the operation department at MOH declared that the density of participants increased during previous years. The country’s capacity to support the health needs of the MG participants is a concern as the health care infrastructure in Iraq has been negatively impacted by ongoing wars and sanctions affecting the country since the 1990s. Health care facilities have been destroyed during violent conflict, some of which have been rebuilt. In addition, Iraq has lost approximately 50% of its workforce of physicians since 1990. There were 34,000 physicians registered in the Iraqi Medical Association in 1990, and only 16,000 physicians were registered in 2008 [10]. The local health care infrastructure in Karbala struggles to meet the needs of the local community and is ill-prepared to serve the health needs of the millions of participants in the Arbaeenia MG. Information on the public health in MGs is needed to support the government in planning for Arbaeenia; however, there have been few formal studies conducted on MGs in Iraq [11]. Adequate preparedness plans are essential for handling medical emergencies during the Arbaeenia MG to prevent causalities and harm to population health.

### Objectives

This study aimed to describe the temporary health care facilities installed and run by the local community and provide health care services to the Arbaeenia MG attendees in Karbala, Iraq, as a first step for mapping available resources.

## Methods

A cross-sectional survey was conducted from December 5, 2014, to December 14, 2014, to describe the health care services provided by the local community to participants in the Arbaeenia MG in Karbala, Iraq. Najaf and Karbala are neighboring governorates, and the distance between Najaf City (capital of Najaf governorate) and Karbala city (capital of Karbala governorate) is 80 km; 22 km are within the boundaries of Karbala governorate. All community-based health care facilities located along these 22 km within Karbala boundaries were chosen in this study. These health care facilities belonged to civil society organizations called Mawakeb Committees. They offered participants voluntary free-of-charge services such as food and bed as well as basic primary health care services. These facilities were visited and examined after the consent was obtained as part of the study.

### Data Collection Tool

A structured questionnaire was used to collect information on the facilities, staff profile, type of services provided, and inventory of medical equipment and supplies. Data were collected by 5 trained data collectors, and information was collected through interviews with the health workers and observations. The questionnaire was pilot tested before the study was implemented.

Through the questionnaire and observation, information was collected on the health care facilities including licensure status given by the MOH, type of building, type of service offered (dispensing medicine, dressing and suturing wounds and injuries, blood pressure and blood sugar measurements, minor operations, and others), documentation and access to ambulance and mobile clinic services, and availability of clean running water.

In addition, the availability and expiration dates of drugs were recorded. Information on the number of doses and duration of antibiotics dispensed was collected, and their expiration dates were verified. Data collectors also obtained information on the health care facility staff, including age, gender, education, and occupation. Health care workers were asked to indicate the type of medical services they offered to the MG participants. Health care workers were also asked if the facility provided services to MG participants on 4 commonly expected illnesses: respiratory diseases, chronic diseases, injuries, and musculoskeletal disorders.

Information was collected on infection control measures and if these measures were used on all event days, including medical waste management; personal protection measures such as washing hands with soap or using alcohol and wearing disposable gloves, face masks, and goggles; and environmental sanitation. An inventory of medical equipment was also conducted.

### Statistical Analysis

The data were entered and analyzed in Epi Info 7 developed by US Centers for Diseases Control and Prevention, Atlanta, Georgia. We estimated the mean age and average score of medical services, drugs, infection control measures, medical services, and medical equipment, in addition to the percentage distribution of health care facility attributes, primary health care services offered by the facilities, availability of drugs, and the sociodemographics of the health workers. We calculated the distribution of the primary health care services by category of health workers, available drugs, available medical equipment, and infection control materials by licensure status of the health care facility. Fisher exact test was used to compare the percentages. A *P* value of less than .05 was considered statistically significant.

## Results

There were 120 health care facilities along the 22 km, toward Karbala from Najaf, that were included in the study. Only 18 (15.0%, 18/120) health care facilities were concrete buildings, whereas the majority (85.0%, 102/120) were tents or caravans. Moreover, 18 facilities (15.0%, 18/120) had MOH licenses, and 87 (72.5%, 87/120) had an identifiable health care facility signage. Presence of mobile clinic or ambulance services in proximity of the surveyed facilities was found only in less than 40% of the outlets.

### Sociodemographic Characteristics

A total of 659 health care workers staffed these health care facilities, 164 (24.9%) for the licensed and 495 (75.1%) for the unlicensed health care facilities. Of the total health care workers, 293 (44.5%, 293/659) were health care professionals. The mean age of the health care workers was 37 years (range: 15-85 years), 84.9% were male (560/659), and 52.5% (349/659) had postsecondary education. Of the 164 health workers staffing the licensed facilities, 101 (61.6%) were health care professionals, compared with 192 (38.8%) professional health care workers in the unlicensed facilities (Table 1).

**Table 1:** Sociodemographic distribution of health care workers working in community-based health care facilities during Arbaeenia mass gathering in Karbala, Iraq, 2014.

Sociodemographic variables		Workers at the facilities		Total (N=659), n (%)
		Licensed facility (N=164), n (%)	Unlicensed facility (N=495), n (%)	
**Sex**				
	Male	132 (80.5)	428 (86.5)	560 (85.0)
	Female	32 (19.5)	67 (13.5)	99 (15.0)
**Educational level**				
	Illiterate	3 (1.8)	20 (4.0)	23 (3.5)
	Primary	4 (2.4)	68 (13.7)	72 (10.9)
	Secondary	47 (28.7)	166 (33.5)	213 (32.3)
	Postsecondary	110 (67.1)	241 (48.7)	351 (53.3)
**Occupation**				
	Physician-pharmacist	8 (4.9)	33 (6.7)	41 (6.2)
	Other health professionals	93 (56.7)	159 (32.1)	252 (38.2)
	Nonhealth staff	63 (38.4)	303 (61.2)	366 (55.5)

### Diseases Categories

Around 97% of health facilities (116/120) provided services for musculoskeletal disorders, 55.8% (67/120) provided services for gastrointestinal disorders, 23.3% (28/120) for respiratory disorders, 28.3% (34/120) for noncommunicable diseases and 16.7% (20/120) for injuries.

### Provided Health Services

Health care workers provided some medical services to the participants of the Arbaeenia MG en route to Karbala (Table 2), which included dispensing drugs (21.87, 370/1692%), dressing and stitching wounds and injuries (18.85%, 319/1692), measuring blood pressure/blood sugar (20.69%, 350/1692), and providing injections (16.67%, 282/1692). The majority of these medical services were provided by paramedical health workers (≥57%), and 81.3% (301/371) of massaging services were provided by nonhealth care workers (Table 2).

**Table 2:** Distribution of provided medical services by type of personnel working at the community-based healthcare facilities during Arbaeenia mass gathering in Karbala, Iraq, 2014.

Medical services	Type of personnel working at community-based healthcare facilities			Total responses (n=1692), n (%)
	Physician-pharmacist, n (%)	Other health care professionals, n (%)	Nonhealth care staff, n (%)	
Dispensing drugs	40 (10.8)	212 (57.3)	118 (31.9)	370 (21.87)
Dressing or stitching	30 (9.4)	203 (63.6)	86 (27.0)	319 (18.85)
Measuring blood pressure or blood sugar	31 (8.9)	212 (60.6)	107 (30.5)	350 (20.69)
Injections	14 (5.0)	197 (69.9)	71 (25.2)	282 (16.67)
Providing massage	2 (0.5)	68 (18.3)	301 (81.1)	371 (21.92)

### Available Medications

In terms of drug availability, analgesics, antispasmodic, antidiarrhea, antibiotics, and ointments (antibiotics, antiallergy, and others) were available in more than 90% of the health care facilities (Table 3). The antihypertensive drugs, oral hypoglycemic medicines, and cough syrups were available in less than 60% of the health care facilities. None of the drugs were expired. There was a wide disparity between the licensed and unlicensed facilities in the availability of all 10 drugs, 39% and 6%, respectively. On a scale of 0-10, the average number of drugs available in the health care facility was 7.3 (73%).

According to the inventory of medical equipment, 72.5% (87/120) of the facilities had a sphygmomanometer, 69.2% (83/120) had a glucometer, and 63.3% (76/120) had a stethoscope (Table 3). Less than 45% of the facilities had the remaining medical equipment: thermometer, tongue depressor, and torch. On a scale of 10, the average number of equipment available in the facility was 5 (50%). The average number varied by the type of facility, 7.4 (74%, 7.4/10) for licensed and 4.6 (46%) for unlicensed facilities.

**Table 3:** Types of drugs and medical equipment available at the community-based health care facilities during Arbaeenia mass gathering in Karbala, Iraq, 2014.

Drugs and medical equipment		Type of facility		Total (N=120), n (%)
		Licensed (N=18), n (%)	Not licensed (N=102), n (%)	
**Drugs**				
	Analgesics	18 (100)	102 (100.0)	120 (100.0)
	Antispasmodics	18 (100)	90 (88.2)	108 (90.0)
	Antiemetic	15 (83)	79 (77.5)	94 (78.3)
	Antidiarrheal	17 (94)	91 (89.2)	108 (90.0)
	Antihypertensive	12 (67)	56 (54.9)	68 (56.7)
	Oral hypoglycemic	12 (67)	59 (57.8)	71 (59.2)
	Antibiotics	16 (89)	96 (94.1)	112 (93.3)
	Ointments	18 (100)	94 (92.2)	112 (93.3)
	Cough syrup	14 (78)	56 (54.9)	70 (58.3)
	Oral rehydration salt	8 (44)	8 (7.8)	16 (13.3)
**Medical equipment**				
	Stethoscope	16 (89)	60 (58.8)	76 (63.3)
	Thermometer	10 (56)	43 (42.2)	53 (44.2)
	Sphygmomanometer	17 (94)	70 (68.6)	87 (72.5)
	Glucometer	15 (83)	68 (66.7)	83 (69.2)
	Light source	11 (61)	23 (22.6)	34 (28.3)
	Tongue depressor	11 (61)	18 (17.7)	29 (24.2)

### Infection control practices

Table 4 shows the infection control practices in the health care facilities. Washing hands with soap and using antiseptic materials were practices observed by all facilities (100.0%, 120/120). Percentage availability and observed differences of the other 9 tracer elements were found to be much higher at licensed facilities and statistically significant, except for using disposable gloves and syringes and presence of a surgical set. Apart from facilities that only dispensing drugs, 90.5% (95/105) of remaining facilities used disposable syringes, 79.0% (83/105) used disposable latex gloves, 44.8% (47/105) used alcohol swabs for cleaning hands, and 41.9% (44/105) had surgical sets. The remaining infection control measures—medical waste disposal box, environmental disinfectants, facemask, and medical coat—were practiced in less than 22% of the facilities (Table 4). Only 5% of the facilities practiced all the infection control measures, 18% for the licensed and 2% for the unlicensed facilities. The average score of the infection control practices was 5.1 on a scale of 10 (51%), 7.8 (78%) for licensed and 4.6 (46%) for unlicensed facilities.

**Table 4:** Availability of infection control measures at the community-based health care facilities during Arbaeenia mass gathering in Karbala, Iraq, 2014.

Infection prevention control measures	Type of facility^a^		Total (N=105), n (%)	*P* value
	Licensed (N=17), n (%)	Not licensed (N=88), n (%)		
Washing hands with soap	17 (100)	88 (100)	105 (100.0)	>.99
Alcohol-based hand rub	17 (100)	30 (34)	47 (44.8)	<.001
Wear disposable latex gloves	16 (94)	67 (76)	83 (79.0)	.08
Use disposable syringes	16 (94)	79 (90)	95 (90.5)	.49
Safety box—medical waste	9 (53)	10 (11)	19 (18.1)	<.001
Receptacle with cover	13 (76)	20 (23)	33 (31.4)	<.001
Use environmental disinfectants	8 (47)	8 (9)	16 (15.2)	.001
Use antiseptic materials	17 (100)	88 (100)	105 (100.0)	>.99
Presence of a surgical set	11 (65)	33 (38)	44 (41.9)	.03
Wear face masks	10 (59)	9 (10)	19 (18.1)	<.001
Wear a medical coat	12 (71)	10 (11)	22 (21.0)	<.001

^a^Clinics that provide only drug dispensing were excluded from the analysis.

## Discussion

### Principal Findings

This study shows that the participants in the Arbaeenia MG had access to some form of health care services through facilities on the route to Karbala. The area covered by the study constituted one-fourth of the distance of the road from Najaf to Karbala, which is considered the main road taken by most of the participants, especially those from southern provinces and who come through the Najaf airport, whereas the remaining three-fourths of the road had a similar distribution to the distance covered by the study. Hence, approximately 460 facilities could be available to serve the 14 million visitors with a facility to population ratio of 1:30,000 [12]. This ratio is below the ratio of primary health care units to population in Iraq (ie, 1:10,000) [13]. Other studies indicate a similar facility-population ratio, that is, 7.4:100,000 [14]. Using a similar assumption as above, the expected number of health care workers available to serve the visits could be approximately 2400, with a health worker to population ratio of approximately 1:6000. Limiting the total expected health workers to expected health professionals of 1065, the adjusted health worker to population ratio would be 1:13,000. This is comparable with the ratio of professional health workers to population in Iraq, that is, 1:15,000. These estimates show that participants in the MG had similar access to health care workers as the general population. The health care facility to population ratio for the visitors is worse than that offered to the Iraq population [15].

The MG participants received limited medical emergency services from the health workers in the health care facilities, including injury management and dispensing drugs. Only one-tenth of the facilities had all the commonly used drugs for the situation. These drugs treat gastrointestinal, respiratory, and common chronic diseases such as hypertension and diabetes, which are common among the participants in Arbaeenia. Similarly, pain killers, antibiotics, and ointments used for multiple illnesses are needed when addressing the health needs of large masses that are at risk of respiratory infections and musculoskeletal pains because of walking long distances [16]; however, 32% of those who dispensed drugs were nonhealth care staff (Table 2), and this malpractice was reported in the Middle East countries [17-19], Saudi Arabia [20], and even in developed countries [21-25]. One-third and one-tenth of the Iraq population aged 25 years and older have hypertension and diabetes, respectively, and drugs to control the 2 diseases were available in almost three-fifths of the facilities [15]. Medical equipment for measuring blood pressure and blood sugar was also available in three-fourth of the health care facilities. For handling medical emergencies, only two-fifths of the facilities had access to mobile clinics and ambulance services for transporting patients to appropriate hospitals, which may not be adequate.

The facilities practiced infection control measures for individual protection, environmental sanitation, and disposing medical waste. However, only one-fifth of the facilities practiced all the identified infection control measures to protect individuals and environment and for proper disposal of medical wastes. The licensed facilities are relatively better than nonlicensed facilities in this area (Table 4). All facilities should practice all the infection control measures required to control the spread of infections and prevent hazards from the environmental and medical wastes. Medical waste disposal should follow special procedures developed by WHO [3,26]; however, less than one-fifth of the health facilities visited had a safe box for disposing medical wastes, which constituted one-half of the licensed facilities. Overall, there were weak medical waste disposal practices and infection control measures. The health facilities can be fertile grounds for spreading infectious diseases to the participants in the Arbaeenia MG and potentially within the region and at a global level.

MGs require well-planned health care services that are executed effectively, including outpatient care services for minor illnesses, care for medical emergencies, and access to ambulance services and designated hospitals for referrals. The temporary health care services for the MG participants traveling to Karbala were set up by local communities without guidance from the MOH. Thus, facility staffing, supply of drugs and medical equipment, and other necessary materials such as infection control materials were established without necessary compliance with MOH standards. The risk of mass causalities is high, and the government should develop an assessment and response preparedness plan that engages the temporary health clinics for future MGs to prevent causalities and health risks. As the event occurs every year, medical education for physicians and other paramedics should include management of medical problems related to MGs.

### Limitations

The main limitation is difficulty in conducting the study among 80 km distances from Najaf to Karbala; therefore, the study was conducted by taking a convenience sample that covered only one-fourth of the distance of the road.

This is the first assessment of community-based health care facilities and resources serving the Arbaeenia MG participants traveling to Karbala. Further assessment of the adequacy, quality, appropriateness, and preparedness to respond to mass causalities is needed.

## Abbreviations

MG: mass gathering
MOH: Ministry of Health
WHO: World Health Organization
